# History of the Inoculation of the Cow-Pox

**Published:** 1799-04

**Authors:** G. Pearson


### *Medical and Physical Journal.*

VOL. I.] APRIL, 1799. [NO. II.

### *History of the Inoculation of the Cow-pox.*

(Continued from page 12 of our last.)

*The following Communication from Dr.* PEARSON *continues the subject down to the date of it, and shews how rapidly the practice increases.*

SIR, *Leicester Square, March* 12, 1799.

I Hope you will pardon me for taking the liberty to inform you, by way of additional evidence to the testimonies I have published on the subject of the cow-pox, that upwards of one hundred and sixty patients, from two weeks to forty years of age, principally infants, have been inoculated since the 20th of January last, by Dr. WOODVILLE and myself, separately.

I shall at present only communicate the following observations:—

I. Not one mortal case occurred.

II. Not one of the patients was considered to be dangerously ill.

III. Although the extreme cases of the severe kind, which ordinarily occur, in the same number of cases, in the inoculated small-pox, did not occur in the above practice; and although many of the patients were even more slightly disordered constitutionally, yet the whole amount of the constitutional illness seemed to be as great as in the same number of patients in the inoculated small-pox.

IV. None of the patients, namely, above sixty, hitherto inoculated for the small-pox, subsequently to the vaccine disease, took the infection.

V. One of the most important facts is, that the local affection in the inoculated part, on the whole, was less considerable, and of shorter duration, than in the inoculated small-pox.

VI. In many of the cases, eruptions on the body appeared, some of which could not be distinguished from the small-pox.

I have ſent the matter of the cow-pox puſtule, on the thread incloſed, in order, if you approve of the inquiry, to inoculate with it; and I intreat you to favour me with the reſult of your trials; but I muſt trouble you to apply the teſt of inoculating with variolous matter ſubſequently to the vaccine diſorder. I have the honour to be,

Sir, your moſt obedient, and moſt humble ſervant,

G. PEARSON.

*P. S.* I am happy to be able to ſtate, that at Berkeley, Dr. JENNER has continued his trials of inoculation with vaccine matter, ſent from London, with good ſucceſs.

I ſhould have given you a more circumſtantial account of the caſes here alluded to, but I think it unneceſſary, as Dr. Woodville has a pamphlet in the preſs on the ſubject.

